# Development of Energy-Selective Surface for Electromagnetic Protection

**DOI:** 10.3390/mi16050555

**Published:** 2025-05-01

**Authors:** Jinghao Lv, Caofei Luo, Jiwei Zhao, Haoran Han, Huan Lu, Bin Zheng

**Affiliations:** 1 State Key Laboratory of Extreme Photonics and Instrumentation, ZJU-Hangzhou Global Scientific and Technological Innovation Center, Zhejiang University, Hangzhou 310027, China; 12431037@zju.edu.cn (J.L.); 0623a85@zju.edu.cn (C.L.); jackokie@zju.edu.cn (J.Z.); 22331200@zju.edu.cn (H.H.); 2 Key Laboratory of Advanced Micro/Nano Electronic Devices Smart Systems of Zhejiang, Jinhua Institute of Zhejiang University, Zhejiang University, Jinhua 321099, China; 3 China Electronics Technology Group Corporation, Ocean Electronics Research Institute Co., Ltd., Ningbo 315100, China; 4 International Joint Innovation Center, The Electromagnetics Academy at Zhejiang University, Zhejiang University, Haining 314400, China

**Keywords:** metasurfaces, energy selective, electromagnetic space safety, adaptive response

## Abstract

Energy-selective surfaces (ESSs) have gained attention as an advanced electromagnetic protection technology. This review discusses the evolution of ESSs, focusing on four key areas: frequency bandwidth expansion, material innovations, functional enhancements, and application diversification. ESSs have evolved from narrowband designs to providing ultra-wideband protection, covering L-band to K-band frequencies. New designs, including non-reciprocal mechanisms and cascaded filters, enhance the shielding efficiency. Material advancements like the use of vanadium dioxide (VO_2_) and micro–nano fabrication techniques have reduced costs and improved performance, enabling higher-frequency applications. Future developments aim to overcome the current limitations, offering a broader bandwidth, higher power tolerance, and faster response times. ESSs play a key role in integrated electromagnetic protection systems.

## 1. Introduction

The rapid development of electronic technology has resulted in the miniaturization, multi-functionality, and low power consumption of electronic devices, but it has also made high-precision integrated circuits more vulnerable. When a part of a system encounters an external electromagnetic pulse (EMP), especially a high-power microwave (HPM) attack, it can lead to severe damage to the device’s functionality and even cause cascading failures, ultimately disabling the entire system [1,2,3]. Therefore, in recent years, electromagnetic protection technology has gained significant attention and been researched globally [4,5].

Metamaterials have garnered substantial attention in electromagnetic control research due to their notable advantages, including high integration capabilities, low insertion loss, and dynamic tunability [6,7,8,9,10,11,12,13,14,15,16,17,18]. These structures are increasingly applied across diverse domains such as wireless communications [19,20,21,22,23,24], reflection and transmission arrays [25,26,27,28,29,30,31], programmable holograms [32,33,34,35], electromagnetic stealth [36,37,38,39], and signal perception and prediction [40,41,42,43,44,45]. Building upon the exceptional performance of metasurfaces, frequency-selective surfaces (FSSs) have been adapted for spatial electromagnetic protection [46,47,48,49,50,51], addressing limitations of traditional approaches such as limiters [52,53,54,55] and absorbing materials [56,57] in multi-threshold shielding. However, conventional approaches often fail to distinguish between high- and low-energy electromagnetic waves, limiting their ability to provide multi-threshold protection within a single device.

**Figure 1:**
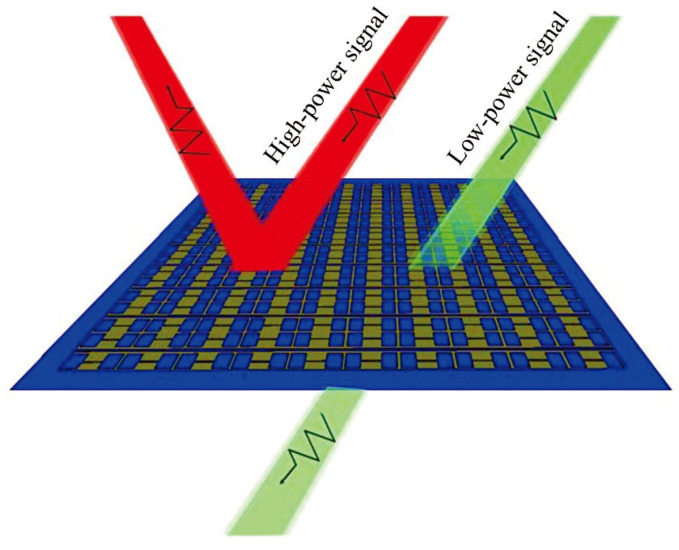
An ESS offers adaptive protection against electromagnetic pulse signals, exhibiting distinct functionalities based on the incident wave’s characteristics. Specifically, when a normal working signal incidents upon the surface, it presents a transparent state, thereby facilitating the seamless transmission of the operational signal. Conversely, upon the incidence of high-power microwave (HPM) signals, the surface transitions to a protective state, effectively serving as an isolation barrier to shield against potential damage. Reprinted with permission from Ref. [66]. Copyright © 2022, IEEE.

In 2009, Liu’s team from the National University of Defense Technology proposed the concept of an energy-selective surface (ESS) to address the limitations of traditional protection methods [58,59,60]. An ESS utilizes nonlinear devices to respond to electromagnetic fields at different power levels, providing adaptive protection against both high- and low-power electromagnetic waves (Figure 1). It exhibits a passband for low-energy signals and a stopband for high-energy signals within specific frequency bands. Based on the principles of frequency-selective surfaces, an ESS not only provides effective shielding from high-power electromagnetic waves but also filters out out-of-band noise, offering dual protection in both the energy and frequency domains.

Over the past decade, ESSs have gained widespread attention and research due to their unique advantages in RF front-end electromagnetic protection. Several theoretical studies have explored the basic principles, working mechanisms, and application effects of ESSs. For example, one study researched the effect of the surface impedance on electromagnetic wave transmission and proposed the concept of a field-induced variable impedance ESS [61]. Another study analyzed the effects of metal grids and diode-loaded metal grids on electromagnetic wave transmission [62]. Additionally, there has been research on the protective mechanism of an ESS from the perspective of electromagnetic fields and current density relations [63]. With the integration of nonlinear components, ESSs have also facilitated progress in simulation methods, such as the periodic boundary method [59], which simulates the electromagnetic frequency domain response of infinite periodic structures but cannot calculate the time-domain or energy-domain characteristics of an ESS. The field–circuit coupled simulation method [60,64,65] effectively solves time-domain response calculations for nonlinear electromagnetic structures. The equivalent circuit method [66], commonly used in simulations, replaces electromagnetic structures with lumped components like inductors and capacitors to simulate the electromagnetic response, but it is only suitable for simple structures with clear electromagnetic coupling paths. For complex ESS structures, precise circuit modeling is more challenging. Liu and Hu [67] have summarized the development of ESSs and established a systematic ESS analysis network.

With further research, ESSs have seen significant progress in their structural design, functionality, application domains, and material selection. This article will review the development of ESSs from four perspectives: frequency bandwidth extension, functional development, application expansion, and material and fabrication process expansion. First, we will briefly review the basic principles of ESSs and analyze the advancements in frequency bandwidth extension and structural design innovations. Next, we will introduce the expansion of ESSs’ functionality, applications, materials, and fabrication processes, providing a comprehensive view of their prospects in the electromagnetic protection field. Finally, we will discuss the future applications and development directions of ESSs.

## 2. Expansion of ESS Bandwidths

An energy-selective surface (ESS) is an electromagnetic protection technology based on field-induced conductive materials, exhibiting electromagnetic environment-adaptive characteristics [58]. An ESS senses the intensity of an electromagnetic field in space, using the spatial electromagnetic energy as an excitation source to dynamically adjust the electromagnetic properties of the material or the impedance characteristics of the structure. This alteration changes the transmission characteristics of electromagnetic waves, ultimately affecting the electromagnetic field distribution in space and achieving effective electromagnetic protection. For equipment that requires protection across different operating frequency bands, an ESS must provide corresponding transmission bands to match the operational requirements of the protected devices. Therefore, the frequency bandwidth extension of ESSs has become an important focus in ESS design and research.

### 2.1. Single-Band ESS

**Figure 2:**
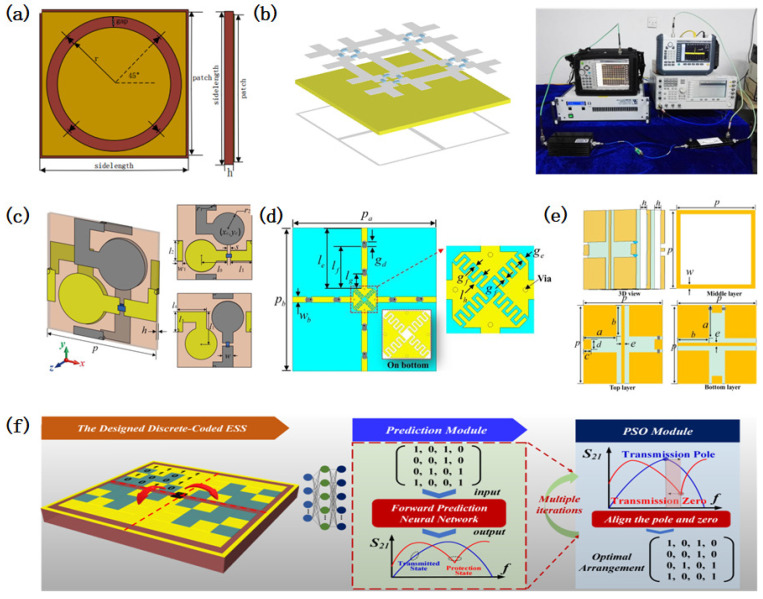
Design of single-band energy-selective surfaces. (**a**) Design of an L-band narrowband energy-selective surface with a ring structure. Reprinted with permission from Ref. [69]. Copyright © 2019, IEEE. (**b**) A micro energy-selective surface based on a tic-tac-toe structure was designed and its insertion loss and protection effectiveness were tested using the waveguide method. Reprinted with permission from Ref. [71]. Copyright © 2019, IEEE. (**c**) A circuit-based dual-resonance ESS design, with its performance tested using the waveguide method. Reprinted with permission from Ref. [73]. Copyright © 2023, IEEE. (**d**) A series-resonant strong-electromagnetic-protection ESS design, which achieved a low insertion loss and high protection effectiveness through an adjustable impedance, formed from diodes and interdigital capacitors. Reprinted with permission from Ref. [74]. Copyright © 2022, IEEE. (**e**) X-band energy-selective surface structure designed using a multi-layer cascade method. Reprinted with permission from Ref. [76]. Copyright © 2023, IEEE. (**f**) Structure and optimization flow chart for an energy-selective surface structure design optimized using machine learning methods. Reprinted with permission from Ref. [83]. Copyright © 2025, IEEE.

Traditional ESS designs primarily focused on aligning the metasurface’s transmission band with the operational frequency band of the protected device, resulting in narrowband, single-frequency solutions with limited angular and polarization stability. Early ESS implementations, constrained by the theoretical frameworks [58,61,62,63], fabrication techniques, and design methodologies [59], were predominantly limited to L-band and S-band applications. For instance, pioneering work by Yang [59] demonstrated the production of an ESS with a 1.3–2.0 GHz transmission band, achieving an insertion loss (IL) of <2 dB and a shielding effectiveness (SE) of >20 dB using voltage-controlled conductive structures. Subsequent improvements by Yi [68] reduced the IL to <1 dB below 1.8 GHz while maintaining a 19 dB SE under high-power microwave (HPM) exposure. Later designs, such as those by Yang [69], targeted specific applications like GPS antennas (1.4–1.6 GHz, IL < 3 dB, SE > 30 dB), highlighting the gradual optimization of ESS performance metrics (Figure 2a).

Advancements in resonant structures, material engineering, and fabrication technologies have expanded ESSs’ operational bandwidths and enhanced their stability. Deng [70] achieved a 33 dB SE near 3.3 GHz, while Wang (Figure 2b) [71] and Chen [72] developed compact ESS units for 2.5 GHz applications. Qin [73] introduced a circuit-based dual-resonance ESS design method and developed an ultra-thin L-band ESS (Figure 2c). Recent studies have further extended ESS capabilities to higher frequencies, including the C-band [74,75], X-band [76,77], Ku-band [78], and K-band [79], with improved angular and polarization insensitivity (Figure 2d,e). For example, Huang [80] proposed a dual-band equivalent circuit model (ECM)-based ESS operating at 3.5 GHz, enabling a tunable stopband resonance, while Wang [81] demonstrated a cost-effective, dual-polarized S-band ESS using symmetric diode configurations. Polarization-insensitive designs, such as the cross-shaped split-ring resonator (CS-SRR) ESS described in [82], leverage fully symmetric geometries to ensure robust performance under diverse polarization conditions. Table 1 shows a performance comparison of different single-band ESSs. It can be seen from the table that with the continuous deepening of ESS research, single-band ESSs have improved in terms of their operating bandwidth, insertion loss, and shielding effectiveness.

Emerging machine learning (ML) techniques are revolutionizing ESS design by addressing inverse electromagnetic problems. Yao [83] introduced an ML-driven approach combining forward-predictive neural networks and particle swarm optimization to autonomously generate ESS configurations with a minimized IL and maximized SE (Figure 2f). This method circumvents the traditional reliance on equivalent circuit models, enabling the rapid prototyping of ultra-thin, wideband ESS structures. Such innovations align with broader trends in metasurface research, where ML is increasingly applied to optimize complex electromagnetic responses [84,85,86,87,88,89,90,91,92,93,94,95,96,97,98].

Single-band ESS technology has evolved from rudimentary narrowband designs to sophisticated, frequency-agile systems with enhanced stability and adaptability. The ongoing integration of data-driven methodologies promises to further accelerate the development of ESS solutions tailored for increasingly diverse and demanding electromagnetic environments.

### 2.2. Multi-Band ESSs

While single-band ESS designs have achieved notable progress, modern electronic systems increasingly require multi-band protection due to their operation across diverse frequency ranges. Multi-band ESS solutions typically employ two strategies, the structural coupling of resonant units to generate multiple passbands and the dynamic tuning of transmission bands based on external stimuli.

**Figure 3:**
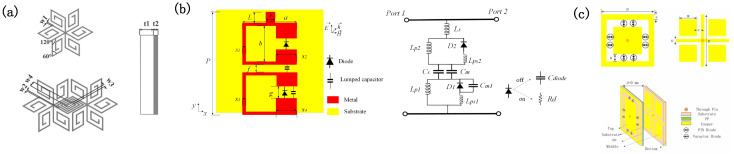
Design of multi-band energy-selective surfaces. (**a**) A multi-band energy-selective surface structure based on a multi-layer cascade of hexagonal spiral structures. Reprinted with permission from Ref. [101]. Copyright © 2016, IEEE. (**b**) A modular LC circuit-based ESS capable of achieving arbitrary band configurations and its LC circuit. Reprinted with permission from Ref. [102]. Copyright © 2022, IEEE. (**c**) A dynamically reconfigurable energy-selective surface structure. Reprinted with permission from Ref. [107]. Copyright © 2024, IEEE.

Early multi-band ESS designs leveraged coupled resonant structures to create distinct passbands. Zhou [99] pioneered a complementary L/S-band ESS with dual passbands, while subsequent studies expanded this concept. Gao [100] modified cross-shaped structures to achieve L/C-band dual-band operation, and Zhou [101] demonstrated a hexagonal spiral ESS with passbands at 3.45–3.95 GHz and 8.3–8.9 GHz (Figure 3a). Hu [102] introduced a modular LC circuit-based ESS capable of achieving arbitrary band configurations, enabling the independent tuning of the insertion loss (IL) and shielding efficiency (SE) per band (Figure 3b). Further innovations include dual open resonant ring structures with nonlinear components, achieving S/C-band operation (2.6–2.8 GHz and 5.4–6.0 GHz) [103], and dual-layer resonant circuits for dual-band ESS designs [104].

Recent advances have focused on dynamically reconfigurable ESSs to accommodate frequency-agile systems. For instance, varactor and PIN diode-coupled structures [105] enable continuous passband shifting, while Zhuo [106] demonstrated an angle- and polarization-stable ESS with a 69.5% tuning range (1.98–3.91 GHz). Xia [107] proposed a tunable ultra-wideband ESS featuring an IL of <3 dB and a center frequency adjustable from 8.31 GHz to 14.83 GHz, achieving a 24.5–31.6% relative bandwidth across states (Figure 3c). These designs highlight the growing emphasis on adaptability in multi-band ESS architectures.

Multi-band ESS technologies have evolved from static coupled resonator systems to reconfigurable platforms, addressing the increasing demand for frequency-flexible electromagnetic protection in multi-functional electronic devices.

### 2.3. Wideband ESSs

The growing complexity of modern electromagnetic systems necessitates universal protection solutions that go beyond the limitations of single- or multi-band ESSs. Wideband ESSs, capable of spanning broad frequency ranges or multiple bands, have emerged as a critical technology for adaptive electromagnetic shielding.

**Figure 4:**
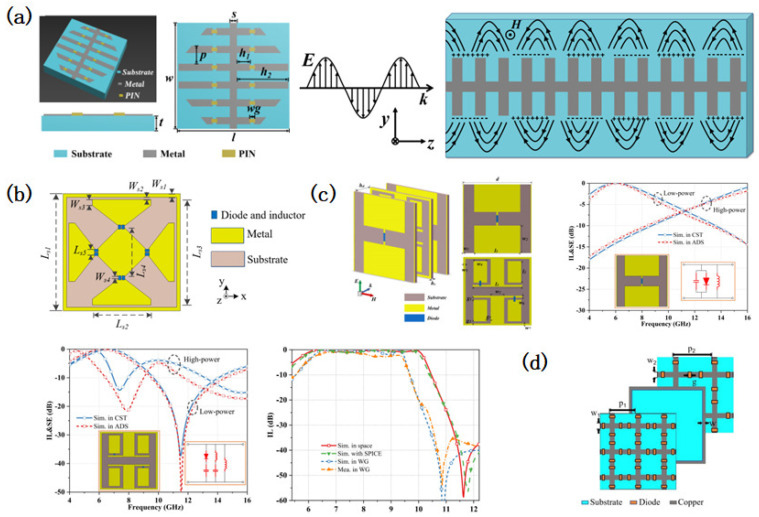
Design of wideband energy-selective surfaces. (**a**) Design of an ultra-wideband energy-selective surface based on a spoof surface plasmon polariton, which increases the protection effectiveness by changing the propagation direction of incident electromagnetic waves. Reprinted with permission from Ref. [110]. Copyright © 2025, IEEE. (**b**) Design of a broadband energy-selective surface based on single-layer structure optimization. Reprinted with permission from Ref. [113]. Copyright © 2021, IEEE. (**c**) A broadband energy-selective surface design based on cascaded high-order filter circuits. The figure shows the performance of a single-layer energy-selective surface and the performance of a multi-layer cascade. Reprinted with permission from Ref. [114]. Copyright © 2024, IEEE. (**d**) A multi-layer cascaded broadband energy-selective surface design achieved a combination of a low insertion loss and high protection efficiency using a band-stop structure design. Reprinted with permission from Ref. [117]. Copyright © 2024, IEEE.

Early wideband ESS designs focused on structural modifications to extend the bandwidth. Hu [61] pioneered a single-resonance-based ESS covering 2.28–3.81 GHz (51% relative bandwidth), while Li [108] demonstrated a dual-layer Y-shaped ESS for L/S-band operation with a low insertion loss (IL) and high shielding efficiency (SE). Subsequent advancements integrated frequency-selective structures and high-frequency diodes, as seen in Wu’s arrow-shaped ESS spanning the L-, S-, and C-bands [109]. Jiang [110] further achieved ultra-wideband adaptive shielding (6.7–10.8 GHz, 46.9% bandwidth, IL < 1 dB, SE > 10 dB), making progress in balancing the bandwidth and performance under high-power conditions (Figure 4a).

Recent studies have leveraged computational tools to enhance the wideband ESS performance. Li [111] combined cascaded resonant circuits with BP neural network optimization, achieving an IL of <1 dB, SE of >40 dB, and 60° angular stability over a 200% relative bandwidth. Similarly, Zhang [112] utilized equivalent circuit modeling and 3D electromagnetic simulations to design an ESS covering 6.7–16.7 GHz with an SE of >26 dB and dual-polarization stability.

Multi-layer designs have proven effective in broadening the bandwidth while maintaining efficiency. Zhou [113] introduced a dual-resonance mesh cross ESS for full S-band coverage, which dynamically switched between bandpass and band-stop states (Figure 4b). Tian [114] developed a quasi-elliptic response ESS with a cascaded three-layer structure, achieving a 6–10 GHz passband with robust out-of-band suppression (Figure 4c). Wu [115] extended this approach to the C–Ku bands using a third-order filter-inspired three-layer model, while Zhou [116] demonstrated a non-resonant multi-layer ESS with a 5.8–9.0 GHz band (IL < 1 dB) and a 22 dB SE under high-power exposure.

Advanced geometries address polarization and angular challenges. Wu [117] proposed a triple-layer ESS with crossed metal strips and diodes (Figure 4d), achieving dual-polarization protection (7.84–23.01 GHz, IL < 1 dB, SE > 27 dB) and exemplifying the integration of structural innovation with wideband performance. Table 2 shows a performance comparison of different wideband ESSs. It can be observed from the table that there are significant differences in the broadband ESS performance among different design methods and multi-layer designs have better broadband ESS performance.

Wideband ESS research has transitioned from focusing on simple structural extensions to sophisticated, algorithm-optimized, and multi-layered architectures. These advancements underscore the potential for universal electromagnetic protection in increasingly complex and dynamic operational environments.

## 3. Expansion of ESS Functions

The functional evolution of energy-selective surfaces (ESSs) has involved a transition from conventional shielding to adaptive, multi-modal electromagnetic management, driven by emerging demands for intelligent protection. A pivotal advancement is the development of a non-reciprocal ESS by Wu [118], which achieves unidirectional high-intensity radio frequency (HIRF) isolation across ultra-wideband frequencies. This design employs an electromagnetic (EM) detector to monitor the forward electric fields, triggering a dynamic transition from transparency to opacity when an HIRF originates from the front, thereby shielding the rear equipment. Crucially, a rear-originating HIRF bypasses this mechanism, ensuring unimpeded outward radiation, a breakthrough for bidirectional electromagnetic control. Further innovations have focused on tunable response thresholds (RTs). Hu [119] optimized the RT using auxiliary structures (ASs) integrated into series LC circuits, enabling precise activation thresholds in ESS arrays. Concurrently, Liu [120] leveraged PIN diode state-dependent characteristics and envelope detection to create an adjustable RT ESS, where a receiving antenna samples the field strength to dynamically modulate shielding activation. Structural reconfigurability has been advanced through biomimetic approaches, exemplified by Guo [121], who utilized origami-inspired folding to adjust the strip density in the x-direction. This mechanically tunable design enhances the TM-mode shielding and angular stability, demonstrating the synergy between geometric adaptability and electromagnetic performance. Additionally, the main functional expansion directions for ESSs include wideband absorption invisible ESSs and ESSs with wideband protection capabilities.

### 3.1. Invisible ESSs

Achieving electromagnetic invisibility [122,123,124,125,126,127] remains a critical challenge for ESSs, as conventional designs exhibit high out-of-band reflection coefficients, rendering systems vulnerable to detection. Recent advancements have addressed this limitation through two primary strategies, broadband absorption and reflection phase manipulation, enabling a stealth functionality without compromising the shielding performance.

**Figure 5:**
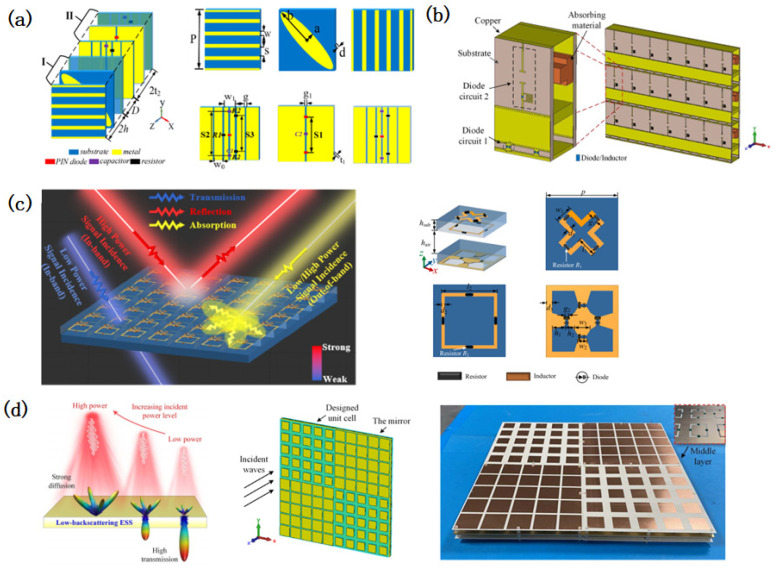
Design of invisible energy-selective surfaces. (**a**) A six-layer cascade invisible energy-selective surface: the top three layers play a role in wave absorption, and the bottom three layers play a role in energy selection. Reprinted with permission from Ref. [129]. Copyright © 2020, IEEE. (**b**) Design of a 3-D absorptive energy-selective surface. Reprinted with permission from Ref. [131]. Copyright © 2021, IEEE. (**c**) An energy-selective surface that achieves invisibility through phase regulation. The upper layer is a frequency-selective surface that achieves phase regulation using varactor diodes, and the lower layer is an energy-selective surface that enables an energy selection function. Reprinted with permission from Ref. [132]. Copyright © 2024, IEEE. (**d**) An energy-selective surface that achieves invisibility through phase control, utilizing 180° phase-shifted reflective units to scatter signals. Reprinted with permission from Ref. [133]. Copyright © 2021, IEEE.

Integrating electromagnetic loss materials with ESS structures suppresses out-of-band reflections. Ran [128] combined frequency-selective surfaces (FSSs) with ESSs using an equivalent circuit model, achieving in-band protection and out-of-band stealth. Yuan [129] introduced dedicated loss layers to traditional ESS designs (Figure 5a), while Gong [130] optimized ultra-wideband (UWB) absorption with low-profile geometries. Early designs like that of Zhou [131] employing rectangular absorbers suffered from a narrow bandwidth and excessive profile height, highlighting the need for advanced material and structural innovations (Figure 5b).Qu [132] further developed a nonlinear absorber switching between transmission–abasorption and reflection–absorption modes, enhancing adaptability (Figure 5c).

Phase-engineered ESS units minimize detectability by scattering incident waves. Zhou [133] proposed asymmetric units with identical transmission but phase-opposed reflections, enabling low-power transmission and high-power backward scattering (Figure 5d). Li [134] utilized 180° phase-shifted reflective units to scatter signals across 3.4–7.0 GHz in the shielding mode, reducing the RCS while maintaining the 4.5 GHz transmission efficiency. These approaches demonstrate the potential of phase manipulation to achieve stealth without using additional absorbers.

### 3.2. Protective ESSs

Protective ESS designs are broadly categorized into low-pass and high-pass configurations based on their frequency-dependent energy response characteristics. Conventional ESS implementations, which integrate PIN diodes into metal grid gaps, face inherent trade-offs between an ultra-low insertion loss (IL) and high broadband shielding effectiveness (SE) at elevated frequencies due to zero-impedance effects and the absence of resonant poles. To reconcile these conflicting requirements, recent studies have adopted cascaded ESS–frequency-selective surface (FSS) architectures. This hybrid approach enables simultaneous signal transmission/reception and an enhanced broadband SE while tailoring passbands to specific devices’ operational frequencies [135,136,137,138,139].

**Figure 6:**
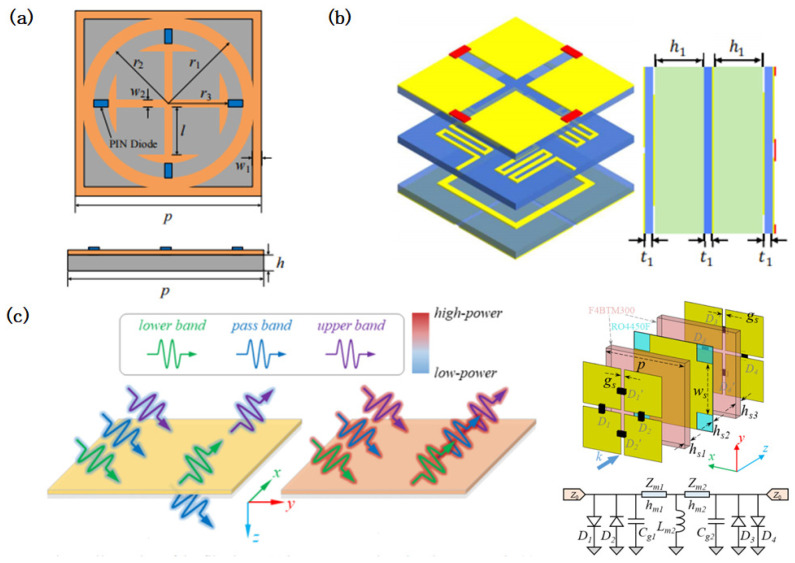
Design of protective energy-selective surfaces. (**a**) A protective energy-selective surface with a single-layer structure design. Reprinted with permission from Ref. [140]. Copyright © 2021, IEEE. (**b**) A schematic diagram of a structure that couples an energy-selective surface and a frequency-selective surface. Reprinted with permission from Ref. [141]. Copyright © 2024, IEEE. (**c**) Schematic diagram of the working principle, structure, and equivalent circuit of a multi-layer cascade protective energy-selective surface. Reprinted with permission from Ref. [142]. Copyright © 2025, IEEE.

Notable advancements include the following:

- Reference [140] (Figure 6a): Optimized single-layer ESS structures delivered a 200% relative bandwidth SE under high-power conditions.
- Reference [141] (Figure 6b): A reconfigurable multi-layer ESS-FSS cascade (one ESS with four FSS layers) achieved a 76.76% fractional bandwidth (1.14–2.56 GHz) with improved out-of-band suppression.
- Reference [142] (Figure 6c): Filtering ESS units based on coupled resonator topologies demonstrated sharp bandpass transitions and an ultra-wideband SE of >30 dB (2.5–6 GHz) at a 40 dBm incident power.

Innovative hybrid designs, such as frequency–energy composite selective surfaces (FECSS) [143], further broaden the protection bandwidths by coupling FSS and ESS functionalities.

## 4. Expansion of ESS Applications

The practical deployment of ESSs requires the careful consideration of the influencing factors, including the design parameters, component selection, and dynamic response characteristics. Hu [144] demonstrated that ESS response thresholds can be tuned via geometric parameter adjustments rather than semiconductor material changes, revealing a direct correlation between the threshold and the quality factor Qp of its equivalent circuit model (ECM); higher Qp values enable lower-power activation. Concurrently, Li [145] analyzed the diode selection impacts on ESS radomes, focusing on the power damage thresholds under high-power inputs, while Han [146] experimentally validated that PIN diode response times critically affect the shielding efficiency, particularly in array configurations. In addition, ESS applications are expanding into three key domains:

- Energy-Selective Radomes (ESRs): Integrating ESSs into radome structures to protect antennas from high-power interference while maintaining signal fidelity.
- Energy-Selective Protection (ESP): Deploying ESSs as adaptive shielding layers in electronic systems for real-time threat mitigation.
- Energy-Selective Antennas (ESAs): Embedding ESS functionalities directly into antenna designs to enable frequency-agile, self-protective radiation systems.

These directions underscore ESSs’ versatility in addressing electromagnetic compatibility (EMC), high-power resilience, and multi-functional integration challenges across aerospace, communications, and defense systems.

### 4.1. Energy-Selective Radomes

Energy-selective radomes (ESRs) represent a critical advancement in the integration of electromagnetic protection with wireless signal transmission, particularly for aerospace and military systems. By selectively blocking high-power interference while preserving the in-band signal integrity, an ESR addresses the dual demands for operational efficiency and electromagnetic resilience. However, integrating energy-selective surfaces (ESSs) into antenna systems introduces challenges related to antenna–radome coupling and performance optimization.

**Figure 7:**
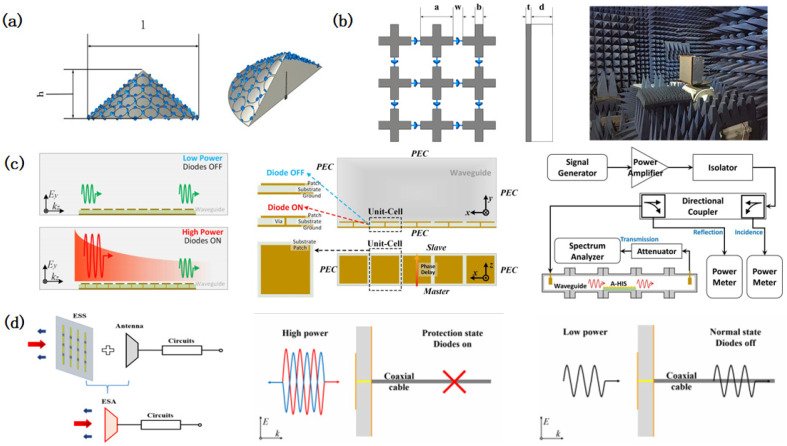
Design of energy-selective surfaces for an expanded range of applications. (**a**) Schematic diagram of a simulation of an energy-selective surface radome. Reprinted with permission from Ref. [151]. Copyright © 2017, IEEE. (**b**) Schematic diagram of energy-selective surface radome structure and experimental test. Reprinted with permission from Ref. [152]. Copyright © 2019, Cambridge University Press. (**c**) Working principle diagram, structural design diagram, and test flow chart for energy-selective protection. Reprinted with permission from Ref. [154]. Copyright © 2021, IEEE. (**d**) Structural design of an energy-selective antenna and a schematic diagram of its working principle. Reprinted with permission from Ref. [155]. Copyright © 2023, IEEE.

Studies have demonstrated ESSs’ compatibility with diverse antenna architectures. For instance, Yi [147] validated the efficacy of ESS-integrated navigation antennas in high-power microwave (HPM) environments, confirming backend circuit protection without compromising signal fidelity. Wang [148] further verified that ESS-based radomes for Beidou navigation antennas maintained the antenna functionality and positioning accuracy, proving seamless integration with existing systems. Advanced methodologies address ESS–radome coupling effects. Hu [149] proposed a Poynting vector-based coupling analysis framework, modeling antennas as transmitters rather than receivers to enhance the evaluation accuracy. Structural innovations include curved ESS (C-ESS) radomes [150], where spherical ESS configurations enable dual-state (transmission/protection) operation validated via patch antenna radiation patterns, extending ESSs’ applicability to curved device surfaces. Zhou pioneered frequency-adaptive ESR solutions. A conical ESS radome integrated with a monopole antenna [151] leveraged circular slot planar ESS simulations to analyze the operational bandwidth and shielding characteristics (Figure 7a). Later work [152] introduced a self-driving frequency-selective radome for L-band microstrip antennas, achieving adaptive shielding through self-triggered diode switching (Figure 7b).

### 4.2. Energy-Selective Protection

Energy-selective protection (ESP) refers to the integration of energy-selective surfaces (ESSs) as modular, pluggable components within waveguide systems to enhance electromagnetic protection. This approach enables the reinforcement of existing electronic systems without structural modifications, offering a cost-effective and adaptable solution for high-power electromagnetic (HPEM) mitigation.

Wu [153] pioneered an ESS protection plug comprising a diode-loaded circuit board sandwiched between ground metal patches. Through ECM analysis, the plug was found to demonstrate a shielding effectiveness (SE) of 13 dB under high-power conditions, validating its utility in waveguide applications. Zhang [154] developed an A-HIS for X-band waveguides, leveraging diode switching to dynamically alter the boundary conditions. When triggered by high-intensity waves, the surface transitioned from a perfect electric conductor (PEC) to a perfect magnetic conductor (PMC), attenuating the incident waves by >10 dB and absorbing 80% of the power within the operational bandwidth (Figure 7c). For L-band applications, Zhang [63] designed an ESS plug with four PIN diodes, achieving an SE exceeding 20 dB. This design underscores the scalability of diode-based configurations for tailored frequency responses. ESP’s plug-and-play architecture simplifies upgrades to legacy systems, particularly in military and aerospace contexts where retrofitting constraints exist. The modularity of ESS plugs allows for rapid deployment in waveguide environments, balancing high-power protection with a minimal insertion loss.

### 4.3. Energy-Selective Antennas

Energy-selective antennas (ESAs) merge electromagnetic shielding with an antenna functionality, enabling adaptive protection against high-power interference while maintaining signal integrity. Central to these designs is the integration of nonlinear components like PIN diodes, which dynamically respond to the incident field strength. For instance, Wang [155] combined microstrip patches with PIN diodes to create a compact ESA (Figure 7d), while Deng [156] embedded S-band diodes into printed dipoles, demonstrating field strength-dependent activation. Lin [157] further simplified this approach for cost-effective protection in high-power environments. Structural innovations, such as Liu’s complementary phase gradient design [50], have expanded the bandwidth in Fabry–Pérot resonator antennas, balancing radiation efficiency and shielding adaptability.

Application-specific ESA solutions address niche operational demands. Fang [158] introduced a novel topology using PIN diodes to reduce aperture coupling under high-intensity radiation (HIRF), validated through simulations and experiments, and Si [159] developed a GPS-focused ESA, shielding against 1.56–1.59 GHz signals without compromising the positioning accuracy.

## 5. Expansion of ESS Materials and Fabrication Processes

**Figure 8:**
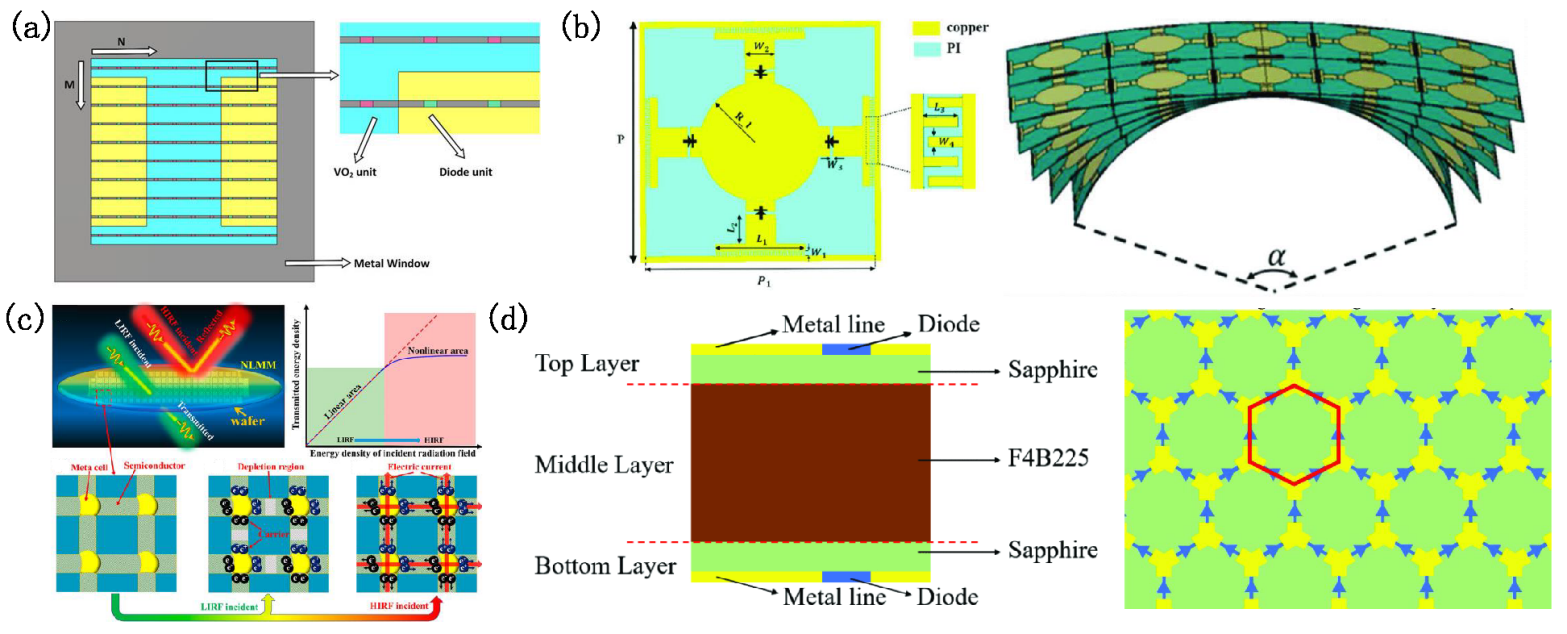
Design of energy-selective surfaces with expanded materials and fabrication processes. (**a**) A new energy-selective surface composed of field-induced impedance conversion material made of VO_2_; using diode units allows VO_2_ to complete impedance transformations faster. Reprinted with permission from Ref. [163]. Copyright © 2023, Wiley Periodicals LLC. (**b**) Schematic diagram of a flexible energy-selective surface structure design and its conformal performance simulation. Reprinted with permission from Ref. [166]. Copyright © 2023, IEEE. (**c**) A nonlinear metamaterial (NLMM) concept based on a metamaterial structure and semiconductor micro–nano processing integrated on the same wafer. Reprinted with permission from Ref. [167]. Copyright © 2023, American Chemical Society. (**d**) Schematic diagram of an energy-selective surface structure using micro–nano technology to achieve high-frequency protection. Reprinted with permission from Ref. [168]. Copyright © 2024, IEEE.

The evolution of energy-selective surfaces (ESSs) has driven innovations in materials and fabrication processes to overcome the limitations of traditional designs reliant on PIN diodes. Vanadium dioxide (VO_2_), renowned for its metal–insulator transition (MIT) under high electric fields [160,161], has emerged as a promising alternative material. However, its high MIT threshold [162] (tens of kV/m) restricts its practical use. To address this, Chen [163] proposed a composite ESS (CESS) combining VO_2_ with diodes, leveraging non-uniform electric fields to reduce the MIT field strength and production costs (Figure 8a).

Conformal ESS designs are critical to allow for flexible applications. Hu [164] employed the synthetic function expansion (SFX) method to analyze cylindrical conformal ESS arrays, achieving efficient radar cross-section (RCS) and field distribution calculations. Xiong [165] developed a flexible ESS to mitigate the biological effects of electromagnetic biological fields, offering a <3 dB insertion loss at 3.5–4.5 GHz and high-power protection from frequencies of 0.1–7.5 GHz. Li [166] demonstrated curvature resilience up to 90° via equivalent circuit modeling, ensuring performance in dynamic environments (Figure 8b).

High-frequency ESSs face challenges regarding diodes’ parasitic effects and fabrication precision. Wu [167] introduced nonlinear metamaterials (NLMMs) through semiconductor microstructure co-integration, enabling adaptive transparency/opacity transitions in microwaves (Figure 8c). Ni [168] achieved a wafer-level ESS with ultra-wideband performance (0–18.62 GHz) using low-junction-capacitance diodes (30 fF) (Figure 8d), while Wu [169] designed a transparent ESS using sapphire substrates, balancing optical clarity and shielding. These advancements underscore the shift toward miniaturized, multi-functional ESSs for next-generation optoelectronic and defense systems.

## 6. Conclusions

Energy-selective surfaces (ESSs) represent a transformative advancement in electromagnetic protection, offering adaptive solutions that combine signal integrity with high-power resilience. This review highlights four pivotal dimensions of ESS evolution: bandwidth expansion, functional diversification, application extension, and material innovation. Early ESS designs, constrained to narrowband operation, have evolved into ultra-wideband systems spanning L- to K-band frequencies through resonant structure optimization, multi-layer architectures, and machine learning-driven design. Functional enhancements now include non-reciprocal isolation, tunable response thresholds, and stealth capabilities, enabled by phase-engineered reflection control and origami-inspired reconfigurability. Their applications extend to energy-selective radomes (ESRs), protection plugs (ESP), and self-shielding antennas (ESAs), addressing critical needs in aerospace, defense, and emerging 5G/IoT systems. Material innovations, such as vanadium dioxide (VO_2_) integration and micro–nano fabrication, further reduce costs and enhance conformal, high-frequency performance.

Despite these advancements, challenges persist. Material limitations, including VO_2_’s high transition threshold and diodes’ parasitic effects at elevated frequencies, hinder scalability. Flexible and transparent ESS designs face manufacturing bottlenecks, requiring cost-effective, large-scale production methods. Additionally, integrating ESSs with existing protection frameworks demands holistic strategies to ensure compatibility and reliability in complex systems.

Future research should prioritize interdisciplinary approaches to overcome these barriers. Advanced materials like graphene-based devices and phase change composites could enhance power handling and frequency agility. Intelligent ESS systems, leveraging AI-driven optimization and real-time adaptive algorithms, promise dynamic threat responsiveness. Hybrid architectures integrating ESSs with energy-absorbing metamaterials and active cancellation technologies may achieve comprehensive front-/back-door shielding. Sustainable fabrication techniques, including 3D printing and wafer-scale integration, could address the miniaturization and optical transparency demands in optoelectronic systems.

As electromagnetic environments grow increasingly hostile, ESSs stand as a cornerstone of next-generation resilience. Their versatility in safeguarding critical infrastructure underscores the urgency of continued innovation. Through interdisciplinary collaboration and advanced engineering, ESSs are poised to secure the future of communication, defense, and aerospace systems in an era of escalating electromagnetic complexity.

## Figures and Tables

**Table 1:** Comparison between single-band ESSs.

Ref.	Working Bandwidth (GHz)	Insertion Loss (dB)	Shielding Effectiveness (dB)
[59]	1.3–2.0	<2	>20
[68]	1.0–2.0	<1	>19
[69]	1.4–1.6	<3	>30
[78]	12–15.2	<3	>20
[79]	19.28–28.24	<2	>30

**Table 2:** Comparison between wideband ESSs.

Ref.	Working Bandwidth (GHz)	Insertion Loss (dB)	Shielding Effectiveness (dB)
[108]	0.0–3.0	<3	>22
[110]	6.7–10.8	<1	>10
[112]	6.7–16.7	<1	>26
[116]	5.8–9.0	<1	>22
[117]	7.84–23.01	<1	>27

## Data Availability

Data sharing is not applicable to this article, as no datasets were generated or analyzed in the current study.
